# Prion-Like Proteins in Phase Separation and Their Link to Disease

**DOI:** 10.3390/biom11071014

**Published:** 2021-07-11

**Authors:** Macy L. Sprunger, Meredith E. Jackrel

**Affiliations:** Department of Chemistry, Washington University, St. Louis, MO 63130, USA; mlsprunger@wustl.edu

**Keywords:** prions, prion-like domains, amyloid, protein misfolding, liquid-liquid phase separation, aberrant phase transitions, chaperones

## Abstract

Aberrant protein folding underpins many neurodegenerative diseases as well as certain myopathies and cancers. Protein misfolding can be driven by the presence of distinctive prion and prion-like regions within certain proteins. These prion and prion-like regions have also been found to drive liquid-liquid phase separation. Liquid-liquid phase separation is thought to be an important physiological process, but one that is prone to malfunction. Thus, aberrant liquid-to-solid phase transitions may drive protein aggregation and fibrillization, which could give rise to pathological inclusions. Here, we review prions and prion-like proteins, their roles in phase separation and disease, as well as potential therapeutic approaches to counter aberrant phase transitions.

## 1. Introduction

Protein folding is essential for life, as proteins serve structural roles, catalyze enzymatic reactions, and transport materials through membranes and cells, among many other functions [1]. Most globular proteins adopt a complex three-dimensional folded structure that is well-defined and associated with specific functions. In contrast, intrinsically disordered proteins (IDPs) do not adopt well-defined structures. Rather, IDPs occupy a highly dynamic ensemble of conformations, and these dynamic structural changes are key to the function of these proteins [2]. While some proteins are fully disordered in their native states, although they may adopt a folded state under certain conditions such as upon interaction with a binding partner, numerous proteins contain both well-folded regions and intrinsically disordered regions (IDRs) [3]. Up to 44% of known proteins are thought to contain at least one IDR of 30 residues or longer [4]. While globular proteins are driven to fold by the propensity of hydrophobic amino acids to pack in the core, disordered sequences have fewer hydrophobic residues, and as such are not driven to collapse by hydrophobic packing [5,6]. Rather, disordered proteins are enriched in hydrophilic and charged residues which promote interactions with aqueous solvent, leading to extended conformations [5,7]. However, disordered proteins or regions can also occupy compact conformations depending on the amino acid sequence and environment [8]. Some intrinsically disordered proteins are enriched in a small subset of amino acids with specific characteristics, which are defined as low complexity domains (LCDs). Examples of LCDs include polyglutamine expansions and the Phe-Gly-rich repeats of nucleoporins [9,10]. With their lower stability and access to more conformations than globular proteins, IDPs, IDRs, and LCDs are also prone to misfolding and aggregation. Indeed, misfolding of such proteins is often associated with diseases such as certain myopathies and cancers, as well as numerous neurodegenerative diseases (NDs) including Alzheimer’s disease (AD), Parkinson’s disease (PD), Huntington’s disease (HD), amyotrophic lateral sclerosis (ALS), and frontotemporal dementia (FTD) [11,12].

Another type of neurological disorder that arises from protein misfolding are prion disorders, such as Creutzfeldt-Jakob disease (CJD) [13]. Prions are infectious proteins that can convert to an alternative conformation [14]. This alternative conformation typically harbors a region that adopts the amyloid fold. Amyloid is comprised of stacked beta sheets which can template the conversion of native protein to the amyloid fold [15]. In addition to driving disease, prions can also promote long-term memory formation in mammals and confer adaptive stress responses in yeast [16,17]. Many DNA- and RNA-binding proteins (RBPs) have prion-like domains (PrLDs) defined by their similar amino acid compositions to yeast prions [18]. Many of these RBPs, such as TDP-43, FUS, and hnRNPA1, also have IDRs or LCDs and have been implicated in NDs such as ALS and FTD [19]. Other amyloidogenic proteins such as amyloid-β (Aβ), tau, and α-synuclein (α-Syn) have some similarities with prions and drive several NDs such as AD and PD [20].

A striking feature of RBPs is their ability to drive liquid-liquid phase separation (LLPS) which is the formation of biomolecular condensates often enriched in RBPs and RNA [21,22]. This LLPS process is driven by multivalent interactions among biomolecules, such as RNA-protein binding and transient interactions between disordered protein sequences [21,22]. Many PrLD-containing RBPs have been found to function as scaffold proteins, meaning they are essential for the droplet structure [23]. PrLDs are suggested to have evolved to promote the recruitment of these problematic RBPs to condensates to preserve their solubility [24]. LLPS droplets frequently form and dissolve in response to cellular signals. However, droplets which do not dissolve properly can undergo an aberrant liquid-to-solid phase transition that leads to protein aggregation and fibrillization [25,26]. Chaperone proteins, which promote cellular proteostasis by regulating protein folding and degradation, can modulate droplet dynamics [27,28,29]. It has been proposed that sequestration of aggregation-prone proteins in liquid-like condensates could be protective [30,31] while an aberrant phase transition could lead to misfolding and formation of pathological inclusions [26]. As such, methods to counter aberrant phase transitions are under development as potential therapeutic targets for several NDs. Current avenues of exploration include the development of nucleic acids, small molecules, and chaperone proteins to modulate the material properties of LLPS condensates [30,32,33]. Here, we review prions and prion-like proteins, their roles in LLPS and disease, as well as possible therapeutic approaches to counter aberrant phase transitions.

## 2. Prions, Amyloid, and Prion-like Domains

Prions, or proteinaceous infectious particles, are a unique class of proteins [14]. A salient feature of prions is their infectious nature, characterized as their ability to spread or propagate between cells and organisms [24,34]. Prion proteins adopt a native, soluble conformation typically associated with a cellular function [14,34]. However, these proteins can convert into a characteristic highly stable prion state. In this prion state, the fold converts to the amyloid conformation, which typically is comprised of cross-beta strands [35]. Here, monomers adopt a beta sheet fold and stack with other monomeric units to form a long unbranched fibril [15]. The amyloid fold is a very stable protein conformation, and as such, amyloid is generally resistant to detergents, heat denaturation, and protease cleavage [36]. Conversion of proteins to the amyloid fold is typically driven by an amyloidogenic region within the protein, which is necessary and sufficient for fibrillization [37,38,39,40]. Conversion to the amyloid fold is thought to be irreversible. Once a small amount of protein accesses the amyloid fold, this small quantity of protein can serve as a template, nucleating and accelerating the conversion of remaining monomeric protein to the amyloid fold [24,34]. Prions are defined as infectious amyloids, but not all amyloidogenic proteins are classified as prions. Prion proteins can serve functional roles, but also cause several devastating disorders which typically affect the central nervous system. A salient feature of prions is their infectious nature, characterized as their ability to spread or propagate between cells and organisms.

### 2.1. Prions and Disease

Prion disorders are unique due to the nature of their transmissibility between organisms [41,42]. Prions were first recognized as nucleic acid free infectious particles in transmissible spongiform encephalopathy (TSE). TSEs are underpinned by a conformation change of human cellular prion protein (PrP^C^) to its pathological state PrP-scrapie (PrP^Sc^) [14]. Prion seeds can spontaneously form from alterations in protein conformation, perhaps just due to ordinary protein conformational dynamics. Conversion to the prion form can also be induced by external stimuli, such as inoculation of a seed [43,44,45,46,47,48]. Different conformations of prions can give rise to distinct prion strains which can lead to varying phenotypes of disease, largely due to differences in neuronal vulnerability [49,50]. For example, as scrapie progresses in goats, most of the animals show an affected nervous system and a subset of goats also display an early scratching phenotype. When goats are inoculated with scrapie brain material from diseased goats, those that received material from goats displaying the scratching phenotype appeared to inherit this scratching phenotype. Conversely, goats inoculated from animals with the disease but without a scratching phenotype became ill, but did not inherit the scratching phenotype [49]. These results indicate that the two phenotypes are due to different strains of the scrapie prion [49]. Various strains of PrP^Sc^ give rise to TSEs in humans including sporadic and familial CJD, fatal familial insomnia, and Gerstmann-Sträussler-Scheinker disease [13,34]. While CJD can be sporadic, familial forms of TSEs are associated with specific mutations in the PrP gene that accelerate its conversion to the prion conformation.

### 2.2. Amyloidogenic Proteins in Neurodegenerative Disorders

Several amyloidogenic proteins have similarities to prions and have been implicated in ND [20]. These include Aβ and tau in AD, polyglutamine expanded huntingtin in HD, and α-Syn in PD [51,52,53,54,55,56,57]. Like prions, these proteins adopt a cross-β fold upon aggregation and are extremely resilient to denaturation. These proteins can also seed their own fibrillization. Here, preformed amyloid fibrils, obtained from aggregate-containing lysate or synthetically formed fibrils, greatly enhance fibrillization. This seeding has been demonstrated to accelerate fibrillization of monomeric recombinant protein in cultured cells and in living animals [20,58,59,60]. As with prions, just a small quantity of amyloid-containing seed is required to robustly initiate amyloid formation [61]. Similar to studies employing PrP strains, it has been demonstrated that both tau and α-Syn can populate distinct conformations and that these strains can be perpetuated and are heritable in cultured cells [62,63]. Furthermore, while inoculations of preformed fibrils in animals initially result in localized pathology, spreading of pathology to distant regions of the brain has been observed in a time-dependent manner [60,64,65,66,67]. For example, localized injection of α-Syn fibrils in mice olfactory bulbs induced α-Syn pathology throughout the brain [68,69,70]. This evidence suggests that Aβ, tau, and α-Syn aggregates can undergo cell-to-cell transmission as the disease progresses. Further evidence of the possibilities for cell-to-cell transmission comes from therapeutic strategies wherein fetal dopaminergic neurons grafted in a PD patient were invaded with tau and α-Syn pathology at the time of autopsy [71]. Disorders such as AD, PD, and HD remain distinct from prion disorders because, while there is evidence of cell-to-cell transmission, there have been no identified cases of disease transmission between people [20,72,73,74,75].

### 2.3. Prions and Amyloids Can Serve Functional Roles

Functional prions and amyloid have been found to serve various roles in humans, as well as in bacteria and in yeast. Examples of functional amyloids in humans include premelanosome protein (PMEL) [76,77,78], peptide hormones [79], cytoplasmic polyadenylation element binding protein 3 (CPEB3) [80,81,82,83], and semenogelins [84,85,86]. PMEL fibrils form exclusively in melanosomes to promote melanin biosynthesis and stabilize organelle structure [76,77]. Cleavage of PMEL to produce amyloidogenic fragments is tightly regulated to only occur in melanosomes, preventing accumulation of PMEL throughout the cell [78,87]. PMEL fibrils catalyze melanin biosynthesis, concentrate melanin, and aid in melanin transport [78]. The conversion of CPEB3 from a soluble translational repressor to an amyloid translational activator is important in long-term memory facilitation in neurological synapses [81,88,89]. SUMOylation of soluble CPEB3 prevents aggregation of its N-terminal glutamine-rich LCD. Neuronal stimulation promotes deSUMOylation and ubiquitination of CPEB3, allowing closely regulated amyloid fibrillization [80,82].

In bacteria, amyloid is a key component of biofilms, which are highly stable heterogeneous matrices [90]. These extracellular structures consist of lipids, polysaccharides, and extracellular DNA in addition to amyloid, and provide scaffolds in which communities of bacteria can reside [91]. Upon taking up residence in these biofilm communities, bacteria can share resources and confer other beneficial traits, which enables rapid adaptation of the community to stressors. Ultimately, biofilms allow bacteria to thrive in harsh environments and acquire resistance to antibiotics [90,92,93].

Much of our understanding of prion biology comes from work in Baker’s yeast, *Saccharomyces cerevisiae* [94]. Yeast have harnessed prions as non-genetic elements of inheritance to allow for rapid adaptation to cellular stress [95,96]. In yeast, the prions [*PSI^+^*] and [*URE3*] are associated with traits that are heritable in a non-Mendelian fashion. These traits are conferred upon structural changes, whereby the endogenous proteins Sup35 and Ure2 adopt the amyloid fold [17,97,98]. Sup35, a translation termination factor, can convert to its prion conformation under stress as an adaptive response [17,99]. On conversion to the amyloid fold, Sup35 no longer serves to terminate translation allowing for stop codon read through, thereby promoting expression of cryptic genetic variations in 3′ untranslated regions. This is thought to allow for rapid adaptation, and therefore a growth advantage under certain conditions [16,95,100,101]. The [*URE3*] prion is associated with nitrogen catabolite repression, thereby allowing yeast to utilize diverse nitrogen sources. However, it has also been suggested that fungal prions can be detrimental to yeast. For instance, certain variants of [*PSI*^+^] and [*URE3*] confer growth defects [102,103]. In regulating prions, yeast employ a native prion disaggregase, Hsp104, which is a hexameric AAA+ ATPase that regulates prion formation, elimination, and propagation [104,105,106,107,108,109].

### 2.4. Prion-Like Domains in Human Proteins

The identification of multiple yeast prions has generated interest in better understanding the full complement of prion proteins. Biochemical screens were performed to identify yeast proteins with prion properties. These screens identified 24 new proteins with similar properties to [*PSI^+^*] and [*URE3*], such as the ability to self-assemble and the heritability of traits [110]. Additionally, the newly identified yeast prions were found to share similar amino acid compositions in their amyloidogenic regions, suggesting an identifiable characteristic of prion proteins. Based on these amino acid biases identified in yeast prions, the PLAAC algorithm was developed to search the human proteome for potential prions [111]. 246 candidate proteins were identified with regions of similar amino acid compositions to those of yeast prions. These domains were termed prion-like domains (PrLDs), accounting for ~1.2% of the nearly 20,000 genes screened [18,111,112,113,114]. Using gene ontology analysis, over half of the proteins identified in this study were predicted to bind DNA or RNA [18,114,115]. Strikingly, following their initial identification as putative prion-like proteins, many of these RBPs were later linked to neurodegenerative diseases such as ALS and FTD [18]. Some of the highest ranked PrLD-containing proteins—such as TDP-43, FUS, TAF15, EWSR1, hnRNPA1, hnRNPA2B1, and ATXN2—are known to form pathological inclusions in ALS and FTD patients [116]. TDP-43 aggregates are also observed in AD, HD, and PD patients [117]. The TDP-43 PrLD is essential for many of its native functions, such as mediating protein-protein interactions, recruitment to stress granules (SGs), and several functions such as miRNA biogenesis and splicing [118,119,120,121,122]. Mutations in TDP-43 and FUS have also been linked to ALS and FTD. Most TDP-43 pathogenic mutations are found in its PrLD and some of these mutations, including Q331K and M337V, have been shown to accelerate its aggregation [123,124]. RNA binding and the PrLD are required for TDP-43 toxicity in model organisms, indicating that both are necessary for misfolding and pathogenesis [125,126,127,128].

Human PrLD-containing proteins display many features similar to those of prions. For instance, these PrL proteins can seed aggregation, form unique strains, induce neurotoxicity, and, in some cases, form amyloid-like fibrils [124,129,130,131,132]. PrLD-containing proteins are presumed to be noninfectious, but examples of cell-to-cell spreading throughout the brain have been observed [133]. Highly saturated concentrations of FUS, TDP-43, and SOD1 in spinal motor neurons suggest that these systems could be poised for LLPS and possibly prion-like propagation [134,135]. Recent studies suggest that while LLPS may be fundamental to TDP-43 function, malfunctioning of TDP-43 LLPS may lead to disease [29,30]. Neurodegenerative diseases typically develop later in life as proteostasis regulation progressively declines, leaving long-lived neurons less able to counter misfolding [136]. Many ALS cases are sporadic, and it is often unclear which mutations are causative. It is possible that conversion to a prion-like species triggers the onset of ALS pathogenesis [137].

## 3. Liquid-Liquid Phase Separation

In early studies investigating changes in nucleoli during the cell cycle, proteins in the nucleolus were found to have unusual properties. Various nucleoli-resident proteins were discovered to form dynamic droplets that could fuse on contact and exchange contents with materials in surrounding regions [138,139,140,141,142,143,144,145,146]. These droplet structures are now classified as membraneless organelles, also known as biomolecular condensates or LLPS droplets. LLPS droplets are formed by the demixing of associative biopolymers upon reaching their threshold concentration [21,22]. This demixing process yields high density droplets that display dynamic liquid-like properties including the capacity to undergo rapid internal rearrangements, exchange with surroundings, coalesce with other compatible droplets, and rebound to spherical shapes upon deformation [26]. Multivalent interactions drive this process, and can include specific RNA-protein interactions, specific protein-protein interactions, and nonspecific weak interactions between protein IDRs or LCDs [21,22]. As such, IDRs and PrLDs are often responsible for driving this demixing process [147,148]. Droplet formation is driven by scaffold proteins and many client proteins can be recruited to these droplets [149,150,151,152,153]. LLPS is believed to be an important physiological phenomenon that can drive essential processes, primarily by increasing the local concentration of specific molecules. For instance, certain biochemical reactions are thought to proceed only upon phase separation of necessary components, and corresponding exclusion of inhibitory particles [21,22]. LLPS can also be a protective process, such as the formation of SGs. For instance, the protein G3BP1 can function as a molecular switch that triggers LLPS in response to free RNA concentrations [154,155].

### 3.1. Role of Prion-Like Domains in LLPS

Many proteins with PrLDs have been found to drive or be recruited to LLPS droplets [23]. PrLDs can interact through weak nonspecific interactions, such as dipole-dipole interactions between polar residues and π-π interactions between aromatic residues [24]. Multivalent binding of RBPs to RNA may also contribute to this LLPS. As many of these PrLD-containing proteins serve essential roles in RNA binding, their condensation in LLPS droplets could allow for preservation of their solubility [151,156]. To enhance the solubility of aggregation-prone RBPs, PrLDs may have evolved to promote controlled condensate formation [24,157]. In support of this idea, RBPs with PrLDs such as Xvelo, RBM14, and hnRNPs have been recognized as scaffold proteins in various physiological membraneless organelles [23,158,159,160,161]. Recent studies have shown that amyloidogenic proteins such as tau and α-Syn, as well as the prions PrP and Sup35, also undergo LLPS [162,163,164,165]. Furthermore, condensate formation by PrP, tau, and α-Syn has been shown to trigger nucleation and fibrillization, suggesting that LLPS of these proteins may be directly linked to disease [162,164,165]. Such findings suggest that LLPS is a common mechanism that promotes aggregation of prions, amyloidogenic proteins, and PrLD-containing proteins.

### 3.2. Aberrant Phase Transitions and Protein Misfolding

**Figure 1:**
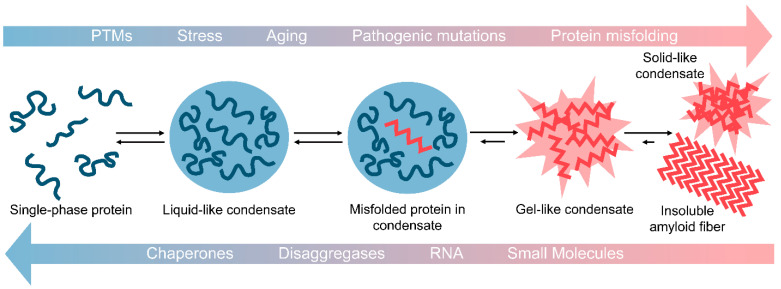
Aberrant phase transitions drive protein fibrillization and aggregation. Many PrLD-containing proteins form liquid-liquid phase separated droplets comprised of protein and often RNA. These droplets have higher protein concentrations as compared to surrounding solutions, which can drive protein misfolding and aggregation. With aging, stress, post-translational modifications (PTMs), and/or pathogenic mutations, these liquid-like condensates can begin to solidify and form gel-like condensates. This process can progress further to produce solid-like condensates of aggregated protein or insoluble amyloid fibrils. Reversal of this process is of great therapeutic interest for many neurodegenerative diseases. Possible approaches to reverse aberrant phase transitions include application of chaperone proteins, protein disaggregases, specific RNA molecules, and small molecules.

In their physiological roles, it is thought that certain condensates frequently form and dissolve in response to external stimuli. However, under certain conditions, such as with aging, mutation, or chronic stress, LLPS droplets may not properly dissipate. When this occurs, the likelihood for misfolding and fibrillization is increased due to the high concentration of aggregation-prone proteins in condensates [25,26,166,167,168]. This can lead to aberrant solidification of the droplet as weak transient interactions within the droplet become more stable (Figure 1) [26]. These resulting highly cross-linked structures can be described as gel- or solid-like [21,22]. In contrast to their liquid-like precursors, gel- and solid-like structures do not display rapid internal rearrangements, do not exchange materials with their surroundings, and cannot readily dissipate [21,22]. Gel-like LLPS structures can also have physiological relevance, as yeast SGs are classified as more gel-like than liquid-like [169]. Some evidence indicates that aberrant LLPS could underpin neurodegenerative disease. Here, certain proteins may phase separate and dissipate repeatedly over time. Yet with aging or other stressors, these liquid-like condensates become more gel and solid-like, ultimately leading to the formation of the solid pathological inclusions observed in patients with neurodegenerative disease [25,26]. Such solid-like structures have been observed experimentally in droplet aging experiments [26].

Droplet dynamics can also be affected in other ways. For example, pathogenic mutations that destabilize proteins can accelerate droplet aging, which has been observed for several pathogenic mutations in the PrLD of TDP-43 [123,124,170]. Also, pathogenic mutations can increase mislocalization of RBPs to RNP granules, causing longer persistence of droplets [113,114,156,171,172]. Additionally, the partitioning of prion-like proteins to LLPS droplets can be modulated by interactions with binding partners and PTMs. For instance, binding of the nuclear import factor Karyopherin-β2 to FUS or methylation of FUS arginine residues both decrease its partitioning into SGs [28,32,173].

An independent disease pathway in ALS also results in impaired LLPS dynamics, emphasizing the importance of maintaining LLPS homeostasis. The most common genetic risk factor for ALS is a hexanucleotide repeat, G_4_C_2_, expansion in *C9orf72* [174,175]. The presence of this expansion results in accumulation of toxic RNA species, production of dipeptide repeat proteins (DPRs) through repeat-associated non-AUG translation, and impairment of nucleocytoplasmic transport [176,177,178,179,180,181,182]. DPRs enriched in arginine (poly-glycine-arginine and poly-proline-arginine) can impair droplet dynamics [174,175,179,181]. These dipeptides can cause spontaneous assembly of SGs and decrease the saturation concentration of hnRNPA1 for droplet formation [181,182]. Arginine-rich DPRs can also trigger aggregation of TDP-43 [183,184]. Thus, multiple ALS pathways are converging on the disruption of condensate formation and dynamics, underscoring the critical importance of maintaining proper LLPS dynamics in neuronal health. Because of the close association between phase separation and proteins that underpin neurodegenerative disorders, there is intense interest in developing therapeutic modulators that restore physiological phase separation.

## 4. Chaperoning Phase Separation

Aberrant phase transitions are believed to underpin protein misfolding and disease; therefore, methods to counter aberrant phase transitions are an emerging area of research which holds great promise. A key goal is to preserve condensates in a liquid-like state and, perhaps more importantly, reverse the conversion to aberrant gel- and solid-like states. To accomplish this goal of preserving aggregation-prone proteins in liquid-like droplets and preventing conversion to pathological states, a range of approaches are being explored, including the development of chaperone proteins, RNA molecules, and small molecule inhibitors.

### 4.1. Protein Chaperones to Counter Aberrant Phase Transitions

Chaperones are a class of proteins that maintain cellular proteostasis by promoting proper protein folding and degradation, as well as preventing and countering protein aggregation [185]. RNP granules and SGs are comprised of several proteins and RNA. Interestingly, these structures are particularly enriched in chaperones such as Hsp40/70, which are likely important for droplet dissolution and maintenance of droplet dynamics [151,186,187,188,189,190]. For example, LLPS droplets of TDP-43 have been found to sequester Hsp70 within the droplet [29]. Hsp70 is also involved in preservation of phase separation in the nucleolus. Here, Hsp70 is required for recovery from heat stress, where it functions to extract and refold misfolded proteins [191]. Impairment of chaperone proteins or limited availability of ATP may hasten droplet aging and solidification [144,151,186].

Certain chaperone proteins have been found to not only prevent, but also reverse aberrant phase transitions. Protein disaggregases are a class of chaperone proteins that can both prevent and reverse protein aggregation. Hsp104 is an AAA+ native yeast protein disaggregase which regulates the assembly and disassembly of prions and also counters protein misfolding especially under stress [104,105,192,193,194,195,196,197]. In yeast SGs and P bodies, Hsp104 maintains droplet dynamics and regulates their dissolution [169]. Although metazoans lack an Hsp104 homolog, it was hypothesized that Hsp104 might be active against proteins that aggregate in human disease due to the conserved fold of amyloid and prion-like proteins. While activity of Hsp104 against many human disease-associated proteins is weak, potentiated variants of Hsp104 have been engineered that can prevent and reverse the misfolding of diverse proteins including TDP-43, FUS, and α-Syn [198,199,200,201,202,203,204,205,206,207,208,209,210,211]. Potentiated Hsp104 variants also displayed therapeutic activity in worm and mammalian cell models of neurodegenerative disease [198,212,213]. Other protein disaggregases have been identified, including Hsp110, Hsp70, Hsp40, as well as HtrA1, NMNAT2/Hsp90, TRIM11, and Karyopherin-β proteins [32,189,214,215,216,217,218]. Nuclear import receptors promote the transport of large proteins across the nuclear pore complex. Karyopherin-β2 (Kap-β2) is a nuclear import receptor that binds to and prevents the aggregation of RBPs including FUS, TAF15, EWSR1, hnRNPA1, and hnRNPA2, many of which accumulate in the cytoplasm in ALS and related disorders [32]. Kap-β2 can also dissolve fibrillized gel-like FUS condensates [32]. Additionally, Importin-α together with Karyopherin-β1 can counter and prevent fibrillization of TDP-43 [32]. It will be important to assess the activity of disaggregases and chaperones to reverse protein misfolding in solidified droplets. Ultimately, small-molecule therapeutics might be designed which stimulate disaggregase activity or promote the interaction of aggregation-prone proteins with disaggregases.

### 4.2. Applying RNA to Modulate Droplet Dynamics

RNA is a common component in biomolecular condensates due to its intrinsically multivalent properties. For instance, paraspeckles and P bodies are membraneless organelles which rely on RNA as a scaffold [219]. More broadly, RNA can have a variety of effects on droplet formation and dynamics. RNA can decrease the threshold concentration for phase separation driven by IDRs, which is the mechanism by which RNA modulates phase separation of hnRNPA1 in SGs and the phase separation of PGL-3 in P granules [25,168,220,221]. In contrast, in some systems, high concentrations of RNA can inhibit phase separation [222]. For example, phase separation of TDP-43 and FUS is inhibited in the RNA-rich nucleus but favored in the cytoplasm where RNA concentrations are lower [222]. Ultimately, this increased phase separation in the cytoplasm can lead to impairment of nuclear import. RNA composition and length have been found to correlate with droplet dynamics [166,223]. For instance, studies on phase separation of the polyglutamine-containing RBP, Whi3, demonstrated that short RNA molecules can decrease droplet dynamics while long RNA molecules have the opposite effect, likely due to RNA entanglement [22,166,223].

RNA molecules are also being explored as a method to alter the formation and dynamics of droplets for prevention of pathological aggregation. As proof of concept, Mann et al. demonstrated that bait RNA molecules binding TDP-43 can prevent aberrant phase transitions and subsequent neurotoxicity [30]. The same affect was not observed with a scrambled RNA control [30]. Additionally, they found that cytoplasmic inclusions of TDP-43 lack mRNA, suggesting that RNA may play a protective role by maintaining dynamic SGs and preventing TDP-43 aggregation [30]. In the future, tailored RNA molecules could be designed against specific therapeutic targets. Additionally, the development of other nucleic acid therapeutics such as antisense oligonucleotides have paved the way for RNA treatments.

### 4.3. Chaperoning Phase Transitions with Small Molecules

Condensates and their associated aggregation-prone proteins present challenges to traditional drug development approaches employing small molecules. These dynamic proteins often lack druggable binding pockets which small molecule therapeutics typically target [224]. As our understanding of phase separation and its corruption has grown, so has our understanding of how small molecules influence condensates, though several key questions remain [225]. To exploit small molecule therapeutics against condensates, it is important to understand how small molecules partition into droplets and how small molecules modulate the stability, dynamics, formation, composition, and other physical properties of condensates. Some small molecules such as 1,6-hexanediol broadly dissolve droplets of varied composition, but such molecules would be expected to disrupt many cellular processes reliant on LLPS [169,226]. Despite the highly dynamic nature of IDPs, small molecules can stabilize substrate-free, monomeric, or multimeric states of IDPs to alter their interactions, which are likely to influence condensate formation and properties [227,228,229]. For example, low concentrations of bis-ANS were found to initiate droplet formation of several proteins, including TDP-43, FUS, and tau, while dissolving droplets at high concentrations [33]. When a small molecule displays phase-dependent interactions with a protein, alterations to the phase diagram can be observed upon addition of the small molecule [230,231,232]. Small molecules can be designed to stabilize or destabilize LLPS assemblies by increasing or decreasing their multivalency, respectively [232]. Small molecules can also alter protein expression levels, such as Synucleozid which decreases α-Syn levels by targeting a regulatory element [233]. Beyond the direct decrease in α-Syn expression levels, such a strategy is also likely to alter condensate formation and offers another mechanism by which to target disordered proteins. Alternatively, a more traditional approach can use small molecule enzyme inhibitors to alter PTMs which govern LLPS droplet formation, composition, and dynamics [234]. Attachment of poly (ADP-ribose) (PAR), a negatively charged biopolymer, to target proteins controls localization and phase separation of targets such as TDP-43 and FUS [31,235]. Inhibiting PAR polymerases will downregulate this modification and limit its function in nucleating TDP-43 and FUS droplet formation [26,31,235,236].

Early studies aimed at drugging condensates with small molecules have proven promising. Studies by Klein et al. demonstrated that anti-cancer drugs such as cisplatin, mitoxantrone, and tamoxifen partition preferentially in nuclear transcription condensates. The drugs were recruited by aromatic and polar properties of the condensates, rather than interactions with specific target molecules [237]. Additionally, oxaliplatin has been shown to disrupt nucleoli through chemical modification of scaffold proteins. The disruption of this condensate led to cell death due to decreased ribosome biogenesis, which explains the mechanism of action of this commonly prescribed anti-cancer drug [238]. In another study, lipoamide and lipoic acid were found to be specifically recruited to SGs in a non-toxic manner and relieved the effects of mutant FUS in several model systems [239]. Although there is much progress to be made in the development of compounds to target phase separation, small molecules have the potential to shape the phase separation landscape.

## 5. Future Directions

There has been great progress in our understanding of the molecular interactions driving biomolecular condensate formation, as well as the mechanisms controlling their properties and regulation. Our understanding of the physiological roles of LLPS, as well as how corruption of LLPS is linked to disease, is also rapidly increasing. Key questions remain in our understanding of the relationship between PrLDs and LLPS. With our improved understanding of this relationship, it has become clear that the role of prions, PrLDs, and amyloidogenic proteins in LLPS must be comprehensively understood in order to understand and ultimately develop effective treatments for protein-misfolding disorders. Utilization of nucleic acids, small molecules, or chaperone proteins are promising approaches to target disordered proteins. No therapeutic treatments currently exist for prion disorders, but improved understanding of prion-like and amyloidogenic proteins has the potential to yield new advances towards the development of effective therapeutics.

## Abbreviations

Aβ, amyloid-beta; AD, Alzheimer’s disease; ALS, amyotrophic lateral sclerosis; α-Syn, alpha-synuclein; CPEB3, cytoplasmic polyadenylation element binding protein 3; CJD, Creutzfeldt-Jakob disease; DPRs, dipeptide repeat proteins; FTD, frontotemporal dementia; HD, Huntington’s disease; IDPs, intrinsically disordered proteins; IDRs, intrinsically disordered regions; Kap-β2, karyopherin-β2; LCDs, low complexity domains; LLPS, liquid-liquid phase separation; NDs, neurodegenerative diseases; PAR, poly(ADP-ribose); PD, Parkinson’s disease; PMEL, premelanosome protein; PrLDs, prion-like domains; PrP^C^, cellular prion protein; PrP^Sc^, scrapie prion protein; PTMs, post-translation modifications; RBPs, RNA-binding proteins; SGs, stress granules; TSE, transmissible spongiform encephalopathy.
